# An oral health education video game for high caries risk children: study protocol for a randomized controlled trial

**DOI:** 10.1186/s13063-015-0754-6

**Published:** 2015-05-28

**Authors:** Ahmad Aljafari, Colm Rice, Jennifer Elizabeth Gallagher, Marie Therese Hosey

**Affiliations:** Division of Population and Patient Health, King’s College London Dental Institute, Bessemer Road, London, SE5 9RS UK; Western Isles Dental Centre, MacAulay Road, Stornoway, Isle of Lewis HS1 2BB UK

**Keywords:** Early childhood caries, Oral health education, Randomized controlled trial, Serious games

## Abstract

**Background:**

Tooth decay is the most common chronic disease of childhood in the world. Many children develop caries early in their lives, and go on to develop further caries and sepsis as they grow up, indicating failure in prevention. As a result, many end up requiring general anaesthesia to undergo treatment for a disease that is completely preventable. Previous studies have suggested that the families of these children need better oral health education as well as better support in implementing healthy practices at home, as they feel impeded by broader life challenges. Parents of these children have suggested utilizing modern technologies, such as the internet, DVDs and video games as methods of delivery of education that might fit in with their busy lifestyles. The aim of this investigation is to assess the acceptability and efficiency of an oral health education video game directed at these children and their families.

**Methods/Design:**

A two-armed phase-II randomized controlled trial will assess a children’s oral health education video game in comparison with verbal oral health education in terms of: family satisfaction, effect on oral health knowledge, and effect on dietary and oral hygiene habits. Up to 110 four- to ten-year-old children, referred for tooth extraction under general anaesthesia due to caries, will be recruited. A sample of 45 participants in each group will be needed to provide 80 % statistical power. The primary outcome measures for this study are: (1) parent and child satisfaction with the intervention, as indicated using a visual analogue scale; (2) improvement in the child’s dietary knowledge measured by a pictorial dietary quiz; and (3) changes in the child’s diet and oral hygiene habits, measured using a children’s dietary questionnaire completed by the parent, and snacking and toothbrushing diaries completed by the child. Measures will be taken at baseline, directly after the intervention, and three months later.

**Discussion:**

This study is a phase-II randomized controlled trial of an oral health education video game for high caries risk children and their families. Few protocols such as this are available in this much-needed research area.

**Trial registration:**

ISRCTN94617251.

## Background

Tooth decay is the most common childhood illness in the world [1]. Early childhood caries is commonly defined as the occurrence of any sign of dental caries on any tooth surface before the age of 6 years [2]. In England, 12 % of children aged three [3], and 28 % of children aged five, have dental caries [4]. The disease can gravely affect the quality of life of these children and their families [5]; furthermore, contracting the disease at an early age puts these children at greater risk of developing caries and sepsis in the future [6, 7]. For a significant proportion, the end result is dental extraction under general anaesthesia, which nowadays is the most common reason for hospital admission in children aged five to nine years in England [8]. Reported general anaesthesia repeat rates are fairly high (25 %), despite rigorous examination and radical treatment [9]. Moreover, many of these children have siblings who have had similar treatment [10]. These findings suggest that there is failure in improving caries prevention in those children and their families postoperatively. Indeed, almost 80 % of parents of these children requested more support in preventing dental caries in their children. Furthermore, 55 % requested that more help with caries prevention be included in subsequent hospital visits [10].

Previous studies suggested that these families display poor oral health knowledge as well as face difficulties in implementing healthy oral habits at home [11]. Given the suggested deficiency in oral health knowledge in these families, provision of oral health advice can be an important part of promoting oral health, and exploration of methods of advice delivery that are effective and acceptable to them is necessary. In a previous study, parents of children referred for extractions under general anaesthesia suggested that different methods for delivering oral health advice can be acceptable, including: websites (64 %), leaflets (63 %), and DVDs (49 %) [10]. In another study, similar findings were reported: (89 %) found leaflets and (67 %) found the internet acceptable methods for oral health advice delivery [12].

The use of video games is a less traditional method of oral health advice that should be considered and explored, as these games might have great potential. Evidence suggests that such games have several advantages over other methods of learning, including: multisensory support, problem-based learning, activation of prior knowledge, immediate feedback, and provision of a social environment involving communities of players [13]. Moreover, video gaming is widely practised nowadays, especially by children. Almost 90 % of American children and teens were reported to play such games on various platforms [14], with the average child aged eight to ten years spending an hour per day playing [15]. In the UK, more than 90 % of six- to nine-year old boys and girls are reported to play such games [16].

Health researchers have realised the potential games may have in delivering health advice. Previous research suggests that using such games to promote a healthy diet carries potential. For example, a study that used a game to promote intake of fruits and vegetables has reported that children had one more serving of fruit or vegetable per day after the intervention and that those with the lowest intake at the baseline benefited the most [17]. Another study assessed the effectiveness of a nutritional education computer package [18] in 8- to 11-year olds in schools compared with traditional methods and suggested that both methods increased the children’s knowledge and that knowledge was retained three months later [19]. A review in 2008 that included 25 studies utilizing such games concluded that they can induce positive health-related outcomes [20]. A more recent review has suggested that amongst 38 studies and a total of 195 health outcomes, video games improved 69 % of psychological therapy outcomes, 59 % of physical therapy outcomes, 50 % of physical activity outcomes, 42 % of health education outcomes, 42 % of pain distraction outcomes, and 37 % of disease self-management outcomes [21]. The reviews also revealed that although these games show the potential to improve health outcomes, most studies are of poor quality and more rigorous randomized controlled trials are needed.

Evidence of the use of video games in oral health education in particular is scarce. A pilot study involving 26 four-year-old nursery children tested a prototype oral health education video game developed by one of the investigators (MTH) and an MSc computer science student [22]. The results suggested no difference in healthy food identification after using the video game, although the sample size was too small to reach significance. Teachers involved in the study viewed the game positively but recommended that it involve more interaction and additional material [23].

In 2008, two of the authors (CR) and (MTH) developed a new prototype oral health education video game. The development process comprised of three steps: first, collection of opinion from experts in the fields of paediatric dentistry, dental public health, nutrition and child education to inform the game’s design. Second, designing the game using the aforementioned input and using Microsoft PowerPoint, and finally, assessment by six six-year-old children to test functionality, child engagement and navigation. The game was named ‘Barney’s healthy foods’, and included an avatar to guide the child through it, as this has been suggested to improve the learning experience [24]. The game was also developed in line with the Scottish National Curriculum, as oral health education programmes that are well integrated into the National Curriculum have been suggested to lead to improvements in children’s oral health knowledge [25]. The game was assessed in a phase-I randomized controlled trial (RCT) that included four- to seven-year-old children in two primary schools in Stornoway, Scotland. The results suggested that this method of oral health education delivery can be as effective as regular paper-based methods in improving children’s recognition of healthy foods [26].

In light of those findings, the current study is a phase-II blind RCT that aims to assess an oral health education video game in comparison with verbal oral health education in terms of family satisfaction, effect on oral health knowledge, and effect on dietary and oral hygiene habits in high caries risk families.

### Aims

#### Primary aim

The primary aims of this study are: (i) to assess the satisfaction of high caries risk children and their parents with delivery of oral health education through an interactive video game, and (ii) to assess the game’s potential to improve the child’s oral health knowledge and dietary and oral hygiene practices.

#### Secondary aim

The secondary aim of this study is to establish proof of concept for the use of interactive video games in delivering oral health education for children.

### Research questions

Would an oral health education video game be an acceptable method to deliver oral health prevention information to high caries risk children and their families? Can the use of such an intervention improve their oral health knowledge and would that improvement be reflected in their oral health practices at home?

### Null hypothesis

The use of a video game to deliver oral health education to children is neither as acceptable nor as effective in improving the child’s oral health knowledge and practices as verbal education delivered by an extended dental nurse or trained health promoter.

## Methods

### Modification of video-game prototype used in phase-I RCT

In preparation for the phase-II RCT, a few modifications of the prototype video game used in the phase-I RCT were necessary. The aim was to update the game in light of the results of the phase-I RCT, newly available technology, dietary guidelines, and the population to be targeted in the phase-II RCT.

**Fig. 1:**
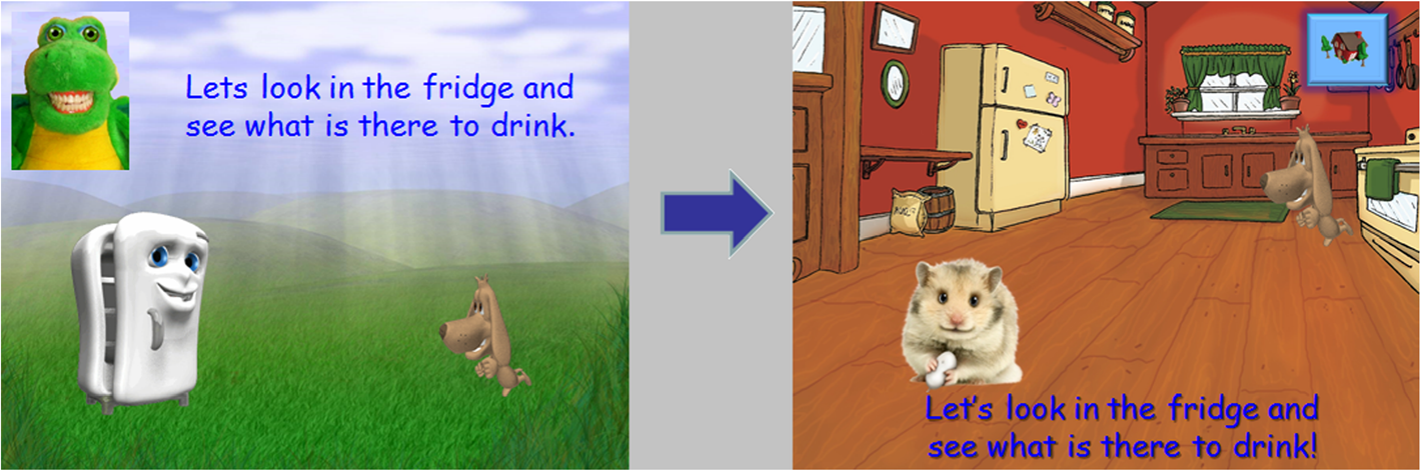
Video-game modification

The research team updated the game content, including the addition of a segment that includes advice on the consumption of fruit juice and another that includes advice on fluoride, as previous findings by the authors have suggested that this cohort of children and their parents received little advice on those two issues [11]. In addition, the game’s graphics were updated, including the introduction of a new avatar (Fluffy the hamster). CrazyTalk7 was used to develop the new avatar, and a new voiceover was recorded and used. Fig. 1 is an example of these updates. Finally, the game’s software was updated to HTML5 (software automatically installed on every computer, Smartphone or tablet) and the game was installed on a tablet (iPad), because they are more popular amongst children [27], can make the gaming experience easier and more interesting [28], and allow for easier set-up in the research location.

Once these modifications were introduced, the researcher (AA) ran the game numerous times to ensure it ran smoothly without any technical difficulties. The game was then further tested by a user group within the pilot study.

### Pilot study

The pilot study aimed to: (i) assess user group interaction with the updated video game and notice any technical difficulties, (ii) assess feasibility of the main study protocol; (iii) assess blinding and randomization methods; and (iv) familiarize all members of the research team with the standard operating procedure.

The pilot study followed the same design, recruitment criteria and methods as the planned phase-II RCT. However, the introduction of blinding and randomization was gradual, so that the research team could be familiarized with the process. Data collection took place in May and June 2013.

Ten participants were recruited. Technical difficulties faced by the users during playing the video game were noted and corrected by the researcher (AA). In addition, the optimum physical setting for the study was determined and the team responsible for the day-to-day operations of the RCT (researcher (AA) and two trained dental nurses, both of whom had a health promotion qualification) became familiar with the recruitment, randomization and blinding processes.

### Phase-II randomized controlled trial protocol

#### Methods and design

This study will be a two-armed blind RCT that will recruit children referred for dental extractions under general anaesthesia, as well as their parents. The participants will be randomized into two groups. In the study group, the child and escort will undergo self-directed ‘play’ about oral health using the video game on an iPad and receive a copy on a DVD to run it on a home personal computer. In the control group, the child and escort will receive verbal oral health advice from a dental nurse who has a health promotion qualification.

The participants will be recruited at the medical pre-assessment clinic at the day surgery unit at King’s College Hospital in London. All children scheduled for a procedure under general anaesthesia attend this clinic approximately two weeks prior to their surgery for a medical evaluation of fitness to undergo general anaesthesia.

#### Ethical considerations

Ethical approval was granted by the National Research Ethics Service Committee London, Dulwich (Reference number: 11/LO/0220), and by the Research and Development Department at King’s College Hospital (R&D Reference number: KCH12-013). Funding was provided through King’s College London PhD funds. Informed consent will be sought from each participating parent or guardian and assent will be sought from the children themselves. Participants will be able to withdraw from the trial at any time and this will not affect access to their treatment at the hospital.

All information disclosed in the study will be kept confidential and participants will not be identifiable in any material published as part of the study in any way. All data are stored without any identifying details. At all stages of research, the data will be stored using a password-protected computer and a secure locked cabinet; and all correspondence between research team members will be conducted using secure email.

#### Participants

The target population will be children referred for extraction of decayed teeth under general anaesthesia. Families will be invited to take part in the study at their child’s attendance at the medical pre-assessment clinic prior to their general anaesthesia appointment.

The inclusion criteria are as follows: the child is four to ten years old, does not have any learning difficulties or medical conditions complicating oral health status and is scheduled for treatment of dental caries under general anaesthesia. A parent or guardian should provide consent and both parent and child should have English proficiency.

The exclusion criteria are as follows: the child is referred for treatment of other dental conditions under general anaesthesia, has learning difficulties or a medical condition affecting oral health, is accompanied by an adult that cannot give consent, or, has been participating in another study. Families where either the parent or guardian or the child do not have sufficient English proficiency to consent or understand the advice delivered will also be excluded.

#### Sample size

The primary outcome measure in this study is user satisfaction with the intervention, assessed using a visual analogue scale. This is constructed as a continuous 100 mm line, with a score at the 0 mm mark indicating complete dissatisfaction with the intervention and a score at the 100 mm indicating complete satisfaction. As far as we know, this is the first study measuring patient’s satisfaction with the use of video games for oral health education. Assuming that our population will have a standard deviation of 25 mm, which is similar to patient satisfaction measured in studies in other fields [29], and aiming to detect a difference of at least 15 mm between the groups to indicate its clinical significance, a sample of 45 participants in each group will be needed to provide 80 % power, at the 5 % significance level, to detect effects of size 0.6 and above. This number of participants is also sufficient based on our calculations using the results of the pictorial dietary quiz used in the phase-I RCT. Anticipating a 20 % dropout rate [30], we intend to recruit approximately 108 participants.

#### Randomization and blinding

A computer-generated simple randomization grid will allocate the participants to the two groups. The randomization process will be overseen by the unblinded researcher (MTH). The allocation of participants will be performed by the two trained dental nurses responsible for applying the interventions to the participants. The researcher (AA) will remain blinded all through data collection and input. The statistician will also be blinded during the analysis. Only after data collection is complete will one researcher (MTH) break the randomization code to input the group allocation within the pre-existing data set and enable between-group analyses. The statistician and lead researcher (AA) will remain blinded.

#### Procedure for recruitment and application of intervention

Each week, the blind researcher (AA) will obtain a list of all children attending the medical pre-assessment clinic for general anaesthesia for dental purposes. Those who are younger than four or older than ten will be excluded.

Next, AA will go through the clinical notes of the remaining children to determine their eligibility to participate. At this stage, children who are having treatment for dental conditions other than caries, or with medical conditions affecting dental health or learning difficulties, and those children participating in other studies can be identified and excluded. All children accompanied by parents or guardians that require an interpreter to provide consent for the general anaesthesia procedure will also be excluded. However, the total number of those children will be recorded. The research will also record the different languages of the non-English speakers. South London is a culturally and ethnically diverse area, and keeping records of the languages spoken by those children and their families will help assess the local area’s future need for versions of the video game in different languages.

On the day of the clinic, AA will approach all potential participants, invite them to take part, and explain the study to them. At this stage, children accompanied by adults who cannot provide consent for them (aunt or uncle, older sibling, *etc*.) and those who do not display enough English proficiency to take part will also be excluded. AA will aim to approach every potential participant matching the inclusion criteria; however, some potential participants will be missed, as they will arrive and leave their appointment while a participant is taking part in the study.

Parents who indicate their agreement to take part will be asked to provide written consent and their child will be asked for assent. AA will then administer three following baseline measures. A pictorial dietary quiz will be completed by the child. This is the same quiz used in the phase-I pilot. (ii) A children’s dietary questionnaire will be completed by the parent. This is a validated measure used to report the dietary habits of children aged 4–16 years [31]. It includes four sections (covering intake of fruit and vegetables, dairy, sweet drinks, and non-core foods). Finally, details of the child’s most recent snack at school will be provided by the child.

After the baseline measures are completed, AA will introduce the participants to the dental nurse and leave, to ensure that he remains blinded to group allocation. The nurse will then allocate the participants to either the video-game group or the control group according to the randomization grid and will apply the intervention accordingly.

The nurse will administer postintervention measures to both groups including: (i) pictorial dietary quiz, completed by the child; (ii) child’s and parent’s satisfaction with intervention on a visual analogue scale; (iii) a booklet that contains a toothbrushing diary and a snack diary, to be returned on the day of the general anaesthesia procedure. The booklet given to children from the study group will also contain a page in which they can write five ‘secret words’ that have been inserted in the DVD version of the game. This will allow us to determine whether participants used the game DVD at home. Children from the control group will have a simple colouring page instead. Finally, (iv) the nurse will ask the parent and child if they have any feedback on the intervention; this feedback will be written down verbatim (qualitative data).

AA will go to the day surgery unit on the day of the child’s general anaesthesia procedure to collect the booklet. He will also call the participating parents three months after their child’s general anaesthesia procedure and offer them a review appointment at the paediatric dentistry department. Upon attending, the parents of both groups will be asked to complete the children’s dietary questionnaire again, to measure any changes in dietary practices at home. The child will be asked to retake the pictorial dietary quiz to assess long-term retention of dietary knowledge. Attendance rates from both groups will be noted.

**Fig. 2:**
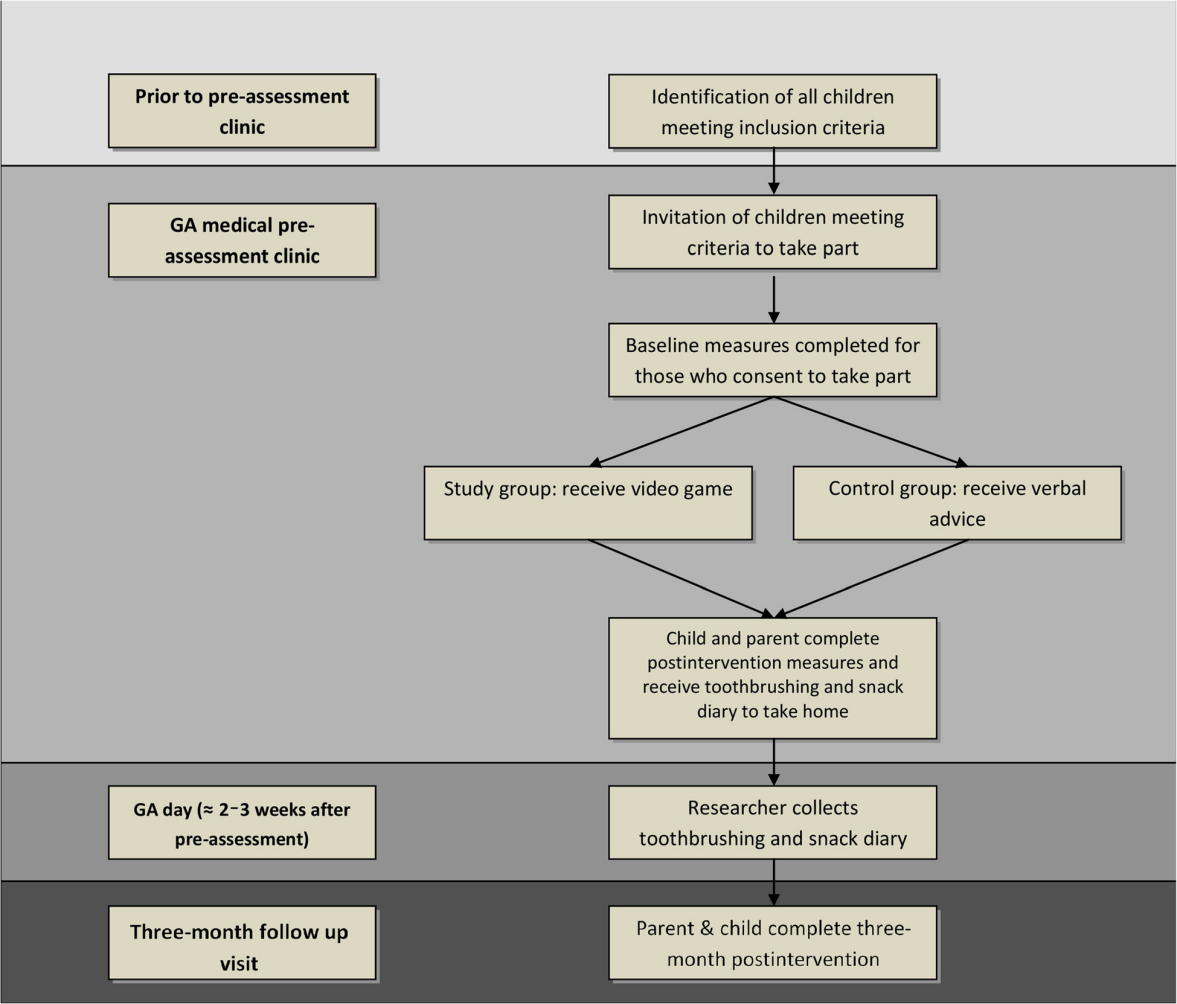
Summary of phase-II RCT process. GA, general anaesthesia

Fig. 2 is a summary of the recruitment process and the measures collected.

#### Outcome measures

**Fig. 3:**
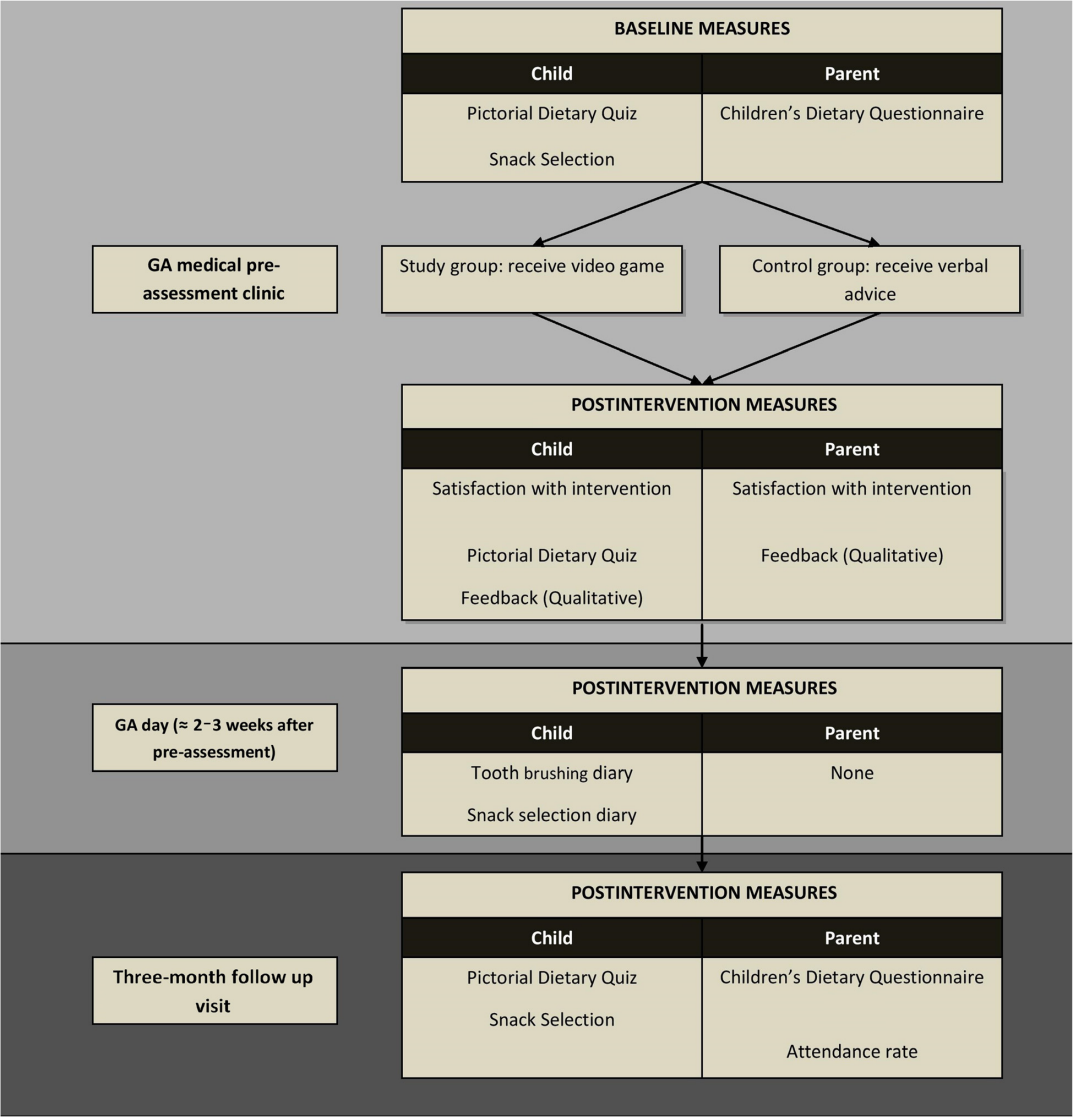
Outline of measure completion. GA, general anaesthesia

Fig. 3 outlines the various measures completed in the phase-II RCT. The primary outcome measures are:

1. Parent and child satisfaction with the intervention, as indicated using the visual analogue scale.
2. Improvement in the child’s dietary knowledge measured by the change in pictorial dietary quiz score taken at baseline, postintervention, and after 3 months.
3. Change in child’s diet at home, measured by children’s dietary questionnaire taken at baseline and after 3 months.
4. Return of the child’s toothbrushing diary showing their level of engagement in toothbrushing, and return of snack diary showing reported healthy snack selection.

The secondary outcome measures are:

1. Parent and child verbal feedback on intervention in the form of qualitative data.
2. Completion of ‘secret words’ sheet by children in intervention group, indicating use of game DVD at home.
3. Attendance rates for review appointment after three months.

#### Statistical analysis

Descriptive statistics for all explanatory variables at baseline will be provided, overall and by study group. Analysis of variance and chi-square tests will be used to highlight any significant imbalance between groups.

Student’s *t* test will be used to compare the two groups’ parent and child visual analogue scale scores, indicating satisfaction with intervention. It will also be used to compare children’s dietary questionnaire, toothbrushing scores, and healthy snack selection scores. Since the children’s pictorial dietary quiz is the only measure taken at three points (baseline, directly after intervention, and three months later), its scores will be analysed using linear multivariate regression.

Verbal feedback provided by parents and children on intervention will be written down verbatim and will be analysed using simple content analysis.

## Discussion

This is the first RCT designed to investigate the use of video games in providing oral health education focused on diet and oral hygiene to this cohort. This study will explore this novel intervention’s acceptability, effectiveness in increasing knowledge, and effectiveness in improving dietary and oral hygiene habits in the short term. This complies with the guidance of the UK Medical Research Council, which recommends that newly developed interventions are not only assessed for effectiveness but also acceptability, as this point is frequently overlooked [32]. Further studies will be necessary, to assess whether such an intervention can lead to sustained changes in dietary and oral hygiene habits and, eventually, positive clinical outcomes. As Nutbeam suggested, evaluating oral health promotion interventions includes four levels: health promotion action (e.g., education), health promotion outcomes (e.g., health literacy), intermediate health outcomes (e.g., health behaviour), and finally, health and social outcomes (e.g., plaque score.) [33].

In our earlier research, parents of children with high caries risk displayed lack of knowledge on the prevention of dental caries [11]. Providing these families with information is important, as knowledge is a vital component in working towards behaviour change, as indicated by social cognitive theory [34]. However, previous evidence suggests that oral health education alone is not enough to achieve clinically significant outcomes [35]. Promoting oral health in such families will require ‘upstream’ action that tackles the social and cultural determinants of the disease supplemented by ‘downstream’ action, focusing on oral health determinants. Nevertheless, delivering oral health advice remains an important part of oral health promotion [36]; the Ottawa charter for health promotion stressed the importance of helping individuals improve personal skills as part of health promotion, and this can be achieved through provision of information, health education and enhancement of life skills in an appropriate manner [37].

Providing oral health education directly to the child might be questioned, since it is assumed that parents are responsible for the families’ oral health practices. However, there is some evidence that children play a role in shaping their oral health practices from a young age; Roberts et al. [38] suggested that children aged seven and older possess some control over their dietary selections in what is commonly known as ‘pester power’. It is important to note that Roberts et al. did not include children younger than 7, but other authors have suggested that children as young as 4 years old have a ‘pester’ or ‘nag’ power over their family’s food shopping [39]. Moreover, Birch and Fisher [40] suggested that even though young children have an innate preference for sugary foods, their dietary preferences are influenced by social and cultural norms, advertising and modelling. Providing children with oral health education through a potentially interesting method, such as video games, might provide them with a positive media influence. In addition, parents are indeed going to be part of the intervention in this study, as they will be required to guide the child through the game. Moreover, parents might learn indirectly, as some evidence suggests that children can transmit knowledge to their parents [41].

Selecting the measures used in this study has been difficult; considering the population targeted. This population is notoriously poor in attending dental clinics regularly [42, 43]. With that in mind, the phase-II RCT is designed to engage families in a positive way about oral health whilst simplifying data collection and reducing the time needed to complete the assessments. Despite this, we anticipate that following participants up at three months might be difficult; hence, we will offer telephone follow-up as an alternative.

The visual analogue scale is a low-burden measure that can be completed quickly and has been validated for use in measuring participant’s satisfaction [29, 30]. There is a risk that participants will feel inclined to give better scores for the nurse providing the control intervention. For this reason, qualitative data will also be collected. Nonetheless, it is recognized that parents might still give socially acceptable answers; thus, the interview approach is important, to ensure that participants feel comfortable in giving their responses.

Measuring the dietary knowledge of children is challenging. A comprehensive review of studies across different health fields reveals how diverse these measures are, with many being designed to measure specific aspects of nutrition knowledge [44]. There is a lack of nutrition knowledge questionnaires for children when oral health is in question. We have chosen to construct a pictorial quiz as this form of questionnaires was suggested as suitable for young children [44], is less likely to be affected by differences in literacy and was also piloted in the phase-I trial. A drawback might be that the quiz will only report recognition of unhealthy items and not knowledge of frequency. It might also be too easy for older children and too difficult for younger ones.

The children’s dietary questionnaire will record changes in dietary practices [31]. This is a 7-day recall dietary questionnaire that is validated and both easy to use and analyse. It contains items measuring consumption of sugary foods and drinks, fits our study design and participant age, and is recommended by the UK National Obesity Observatory [45]. However, it might have poor correlation for some items and has not been yet validated in the UK. Therefore, some of the food items have been altered to UK English to suit the population studied. Children’s snacking and toothbrushing practices will be measured using diaries. Diaries can keep the child involved but these self-reported measures might not be reliable and families might just record the ‘correct’ rather than the real answer [46]. We also anticipate that completion and return rates might not be ideal. Therefore, in this study, story books will be given as prizes to motivate the children, and text messages will be used as reminders for parents. Prepaid envelopes will be provided to those who do not bring the diary back in person, so that they can mail it.

We will not be able to approach all children attending for dental extraction of decayed teeth under general anaesthesia, as many might attend and leave the pre-assessment clinic while the researcher is in the process of applying the intervention to another child. However, the total numbers of those attending, approached and providing consent will be recorded, to reduce bias. Also, it is worth noting that the area served by King’s College Hospital is highly culturally diverse, with many immigrant families [47]. Families without sufficient English language skills will be excluded. These families’ usual languages will be recorded for future reference and can be considered in future versions of the video game.

## Trial status

The phase-II trial commenced in October 2013 and the process of recruitment is still ongoing.

## Abbreviation

RCT: Randomized controlled trial
